# Dosimetric accuracy of delivering SBRT using dynamic arcs on Cyberknife

**DOI:** 10.1002/mp.14090

**Published:** 2020-03-03

**Authors:** James L. Bedford, Simeon Nill, Uwe Oelfke

**Affiliations:** ^1^ Joint Department of Physics The Institute of Cancer Research and The Royal Marsden NHS Foundation Trust London SM2 5PT UK

**Keywords:** arc therapy, delivery accuracy, SABR, SBRT, VMAT

## Abstract

**Purpose:**

Several studies have demonstrated potential improvements in treatment time through the use of dynamic arcs for delivery of stereotactic body radiation therapy (SBRT) on Cyberknife. However, the delivery system has a finite accuracy, so that potential exists for dosimetric uncertainties. This study estimates the expected dosimetric accuracy of dynamic delivery of SBRT, based on realistic estimates of the uncertainties in delivery parameters.

**Methods:**

Five SBRT patient cases (prostate A — conventional, prostate B — brachytherapy‐type, lung, liver, partial left breast) were retrospectively studied. Treatment plans were produced for a fixed arc trajectory using fluence optimization, segmentation, and direct aperture optimization. Dose rate uncertainty was modeled as a smoothly varying random fluctuation of ± 1.0%, ±2.0% or ± 5.0% over a time period of 10, 30 or 60 s. Multileaf collimator uncertainty was modeled as a lag in position of each leaf up to 0.25 or 0.5 mm. Robot pointing error was modeled as a shift of the target location, with the direction of the shift chosen as a random angle with respect to the multileaf collimator and with a random magnitude in the range 0.0–1.0 mm at the delivery nodes and with an additional random magnitude of 0.5–1.0 mm in between the delivery nodes. The impact of the errors was investigated using dose‐volume histograms.

**Results:**

Uncertainty in dose rate has the effect of varying the total monitor units delivered, which in turn produces a variation in mean dose to the planning target volume. The random sampling of dose rate error produces a distribution of mean doses with a standard deviation proportional to the magnitude of the dose rate uncertainty. A lag in multileaf collimator position of 0.25 or 0.5 mm produces a small impact on the delivered dose. In general, an increase in the PTV mean dose of around 1% is observed. An error in robot pointing of the order of 1 mm produces a small increase in dose inhomogeneity to the planning target volume, sometimes accompanied by an increase in mean dose by around 1%.

**Conclusions:**

Based upon the limited data available on the dose rate stability and geometric accuracy of the Cyberknife system, this study estimates that dynamic arc delivery can be accomplished with sufficient accuracy for clinical application. Dose rate variation produces a change in dose to the planning target volume according to the perturbation of total monitor units delivered, while multileaf collimator lag and robot pointing error typically increase the mean dose to the planning target volume by up to 1%.

## 1. Introduction

The Cyberknife system (Accuray Inc., Sunnyvale, CA) has shown itself to be a valuable device for treating patients with stereotactic body radiotherapy (SBRT).^1^, ^2^, ^3^, ^4^ The short‐waveguide 6‐MV flattening‐filter‐free linear accelerator is mounted on a robotic arm and is equipped with either a series of circular collimators, a variable circular diaphragm, or a multileaf collimator (MLC).^5^, ^6^, ^7^ The MLC is widely used to allow treatment of larger tumors using fewer monitor units.

The Cyberknife currently delivers radiation from a number of static locations around the patient in a step‐and‐shoot arrangement.^8^ However, a number of studies have demonstrated the potential for reduction in delivery time by the use of dynamic arc delivery, similar in nature to volumetric modulated arc therapy (VMAT), although from noncoplanar orientations as opposed to the more common coplanar arcs used for VMAT. For example, Kearney et al.^9^ describe a noncoplanar arc optimization algorithm for Cyberknife with a circular collimator. They also describe an optimization method for producing dynamic arcs on the Cyberknife with MLC, using direct aperture optimization.^10^

Simultaneously, data are beginning to emerge on the accuracy of the Cyberknife system. Yang et al.^11^, ^12^ describe a calibration procedure for the ArcCheck quality assurance device (Sun Nuclear, Melbourne, FL) that converts between a spatial error and a dosimetric error, so that the device can be used to measure the pointing accuracy of the robot. Wang et al.^13^, ^14^ use a scintillator and charge‐coupled device camera to record a pair of spots located on the beam axis and calculate from these spots the position of the beam to an accuracy in the order of 0.1 mm. With these data, it is possible to estimate the geometric accuracy of a dynamic delivery, with a view to determining the dosimetric performance.

**Figure 1:**
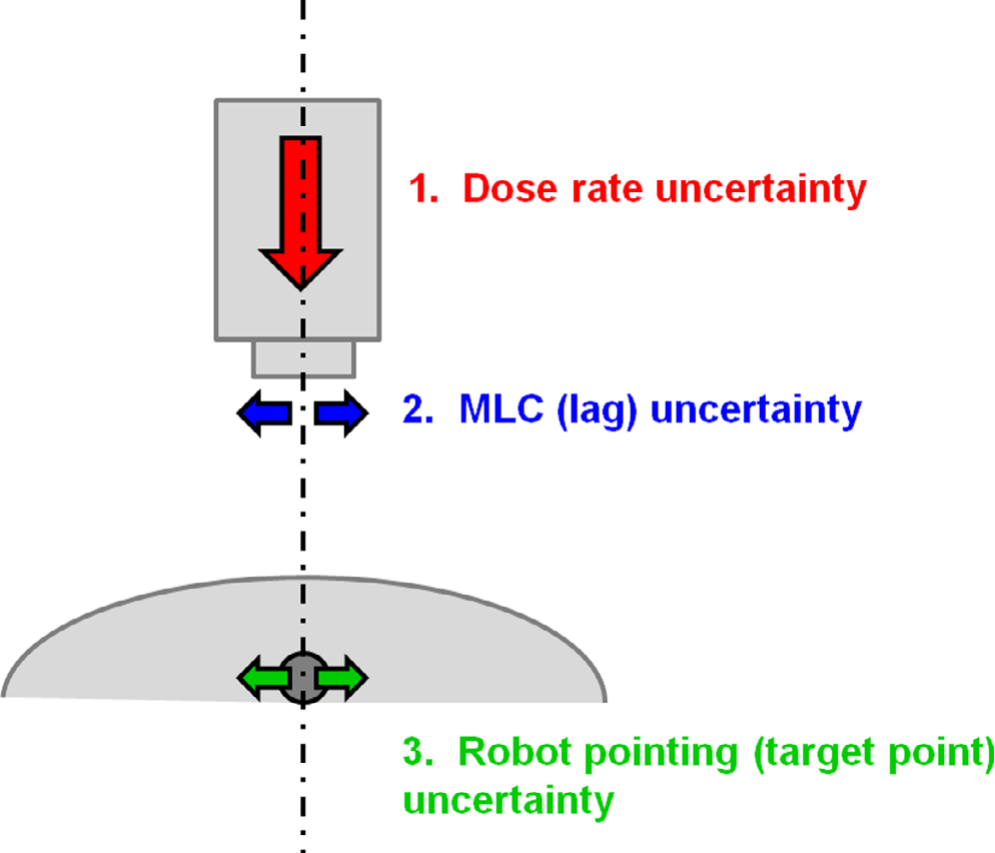
Schematic diagram of the uncertainty model used to estimate the dosimetric accuracy of dynamic arc delivery. [Color figure can be viewed at http://wileyonlinelibrary.com]

This study therefore investigates the performance of arc delivery using the Cyberknife with multileaf collimator, for the case of SBRT. The choice of trajectory is a key aspect of arc delivery, but as trajectory selection is an extensive subject, the reader is referred to previous studies for details.^8^, ^10^, ^15^ In the present study, a previously investigated arc trajectory^15^ is used to provide suitable dynamic baseline plans in the known range of collision‐free operation of the Cyberknife robot. Based on published data and other estimates from Accuray, the expected variations in dose rate, leaf positioning, and robot target position are established and incorporated into models of these parameters (Fig. 1). The consequent impact on the dose distribution is calculated for several cases using these models. Beam delivery between control points is included in the model so as to give the most accurate possible estimation of the dose distribution.

## 2. Materials And Methods

### 2.1. Patient cases

Four patient cases were retrospectively investigated in this study, as follows:

- Prostate A, planned for a homogeneous distribution of dose for a prescription of 36.25 Gy in five fractions according to RTOG 0938.^16^ PTV volume 113 cm^3^.
- Prostate B, the same case planned for a brachytherapy‐style dose distribution using a prescription of 38.0 Gy in four fractions.^17^, ^18^ PTV volume 88 cm^3^.
- Lung, a central lesion prescribed to 50.0 Gy in five fractions according to RTOG 0813.^19^ PTV volume 14 cm^3^.
- Liver, prescribed to 42.75 Gy in three fractions.^20^ PTV volume 28 cm^3^.
- Left partial breast, prescribed to 35.0 Gy in five fractions according to RTOG 0413.^21^ PTV volume 89 cm^3^.

An SBRT technique was used in all cases, with at least 95% of the planning target volume (PTV) being required to receive the prescribed dose. The plans used a fixed isocenter, located at the center of the PTV.

### 2.2. Arc trajectory

**Figure 2:**
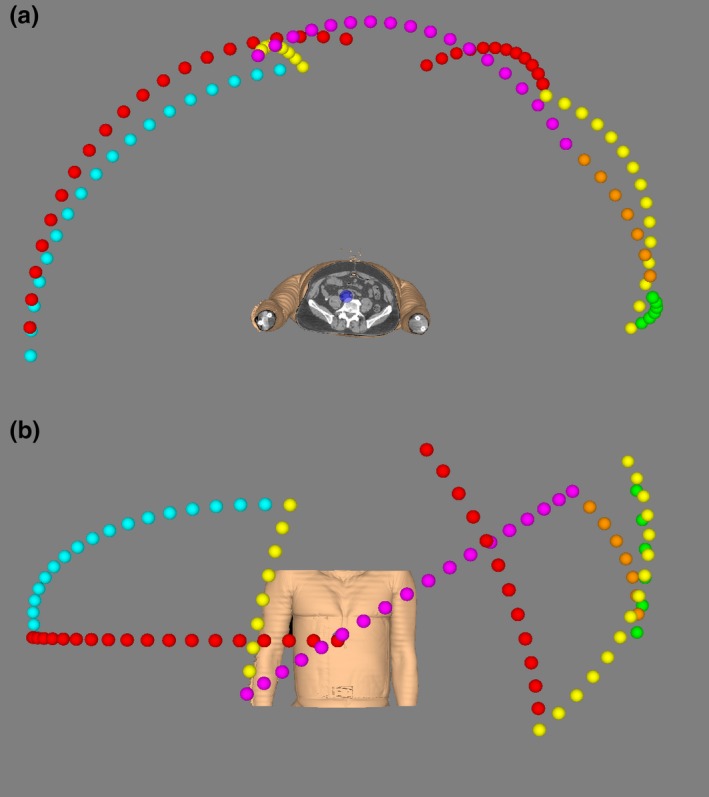
The trajectory used for the dynamic arc plans, illustrated for the liver case. The points give the positions of the radiation source. (a) view from inferior, (b) view from anterior. [Color figure can be viewed at http://wileyonlinelibrary.com]

The arc trajectory used for this study is shown in Fig. 2. It was a purpose‐made path, distinct from the standard body path used by Cyberknife, and using different node positions. It consisted of 104 nodes, or control points, with a spacing of 5° in robot orientation. The trajectory was designed by an Accuray heuristic to give a uniform coverage of the space around the patient, while respecting hardware constraints on collisions and cabling. The performance of the arc in comparison with the standard body path was previously investigated.^15^

### 2.3. Dose calculation and optimization scheme

Dose was calculated as:

(1) $$d_{i}=\sum_{j}d_{\mathrm{ij}}w_{j}$$

where *d_i_* was the dose at voxel *i* in the patient model, *d_ij_* was the dose contributed by beamlet *j* to voxel *i*, and *w_j_* was the beamlet weight. The dose calculation was based on an Accuray‐supplied pencil‐beam algorithm embedded into an in‐house computation and optimization framework. The commercial planning system for Cyberknife was not used in this study. The dose‐influence matrices, *d_ij_*, of Eq. (1) were calculated by using this algorithm to calculate the dose for a series of bixel‐sized fields. The dose grid was 2 × CT pixel size in the transaxial direction and CT slice spacing in the longitudinal direction. Dose voxels which received less than 0.015% of the maximum dose of each *d_ij_* component were neglected so as to minimize the size of the dose matrices.

Plans were produced for all of the cases using a three‐step optimization scheme which produced fluence maps, then sequenced these into deliverable apertures, before performing direct aperture optimization. The resolution of the fluence map was 7.7 mm × 5.0 mm at a nominal source‐axis distance of 800 mm, the 7.7 mm being equal to two leaf widths so that the MLC leaves could be paired.^8^ This was a practical feature designed to reduce the number of fluence bixels and hence reduce memory requirements and increase optimization speed. The optimization minimized an objective function, *F*, defined as:

(2) $$F=\sum_{k}f_{k},$$

where the indices, *k*, referred to individual anatomical structures, each with objective value *f_k_*:

(3) $$f_{k}=a_{k}{\left[d_{i}^{\min}-d_{i}\right]}_{\geq 0}^{2}\;+\;b_{k}{\left[d_{i}-d_{i}^{\max}\right]}_{\geq 0}^{2}.$$

In this equation, the variables *a_k_* and *b_k_* referred to the importance factors for structure *k*. Fluence was optimized using the L‐BFGS optimization scheme, which was a standard gradient descent method using a recursion relation for calculation of search directions, so as to avoid having to store a large and memory‐intensive inverse Hessian matrix.^22^

A fluence map was produced by the optimizer for every third beam orientation. After fluence optimization using 40 iterations, the plan was sequenced using a well‐established sequencer,^23^ with three apertures being used to account for each fluence map. Two of these apertures were redistributed to the beam orientations either side of that for which fluence was optimized. The L‐BFGS method was again used for the direct aperture optimization, with each aperture shape being used to define which fluence pixels were active, and then with the corresponding bixel doses being used to calculate dose and search directions.^22^, ^24^ The optimization itself was therefore based on dose calculated by summation of bixel doses.

During the direct aperture optimization, the method of Christiansen et al.^25^ was used to model the motion of MLC leaves between control points. The motion of one leaf in a leaf pair produced a fluence ramp in one direction, and the motion of the opposing leaf produced a ramp of fluence in the other direction, and the total fluence was calculated as the difference between the two fluence patterns.

The direct aperture optimization also took into account the delivery constraints of the Cyberknife.^15^ The approach taken was that the robot speed was given first priority, defining the delivery time for the arc. The robot speed was taken as 60 mms^−1^, equivalent to the slowest speed observed in practice, so that the time to traverse 5° of arc was calculated to be 1.5 s. The MLC movement was then limited so that the time for MLC motion should not exceed the time taken by the robot to make its movement. The MLC leaf speed was taken to be 33 mms^−1^, which was slightly faster than in current clinical practice, but a speed which was advised by Accuray to be achievable using the current generation of hardware. In the 1.5 s taken for the robot to traverse between nodes, the MLC leaves were therefore able to move 50 mm, and this limit was used by the optimizer. There was no limit to the minimum monitor units per segment, implying that the beam could be paused if required. There was also no limit on the maximum monitor units per segment, which meant that if the time for delivery of the monitor units was large, it could exceed the time taken for the robot motion. In practice, the robot would slow down in this situation. Further details of the optimization scheme are given elsewhere.^15^

After the optimization, dose was recalculated using the Accuray dose calculation in the local computational framework, based on complete apertures rather than on the sums of bixel doses used during the optimization. The dose grid resolution used for this recalculation was the same as for the bixel‐based dose calculation used during optimization. The recalculated aperture dose had a very similar relative dose distribution to the original bixel‐based dose produced by the optimizer, but was seen to be offset by a case‐specific factor of several percent. This resulted from the difference between calculating dose due to MLC‐shaped apertures and due to collections of individual 7.7 mm × 5 mm apertures. The aperture dose was actually the more accurate dose, but as the treatment plans had been created and clinical constraints had been met using bixel‐based doses, the recalculated plan was renormalized to return the dose approximately to the bixel‐based dose distribution, so that the clinical constraints were met as closely as possible. Since the difference between the aperture‐based and bixel‐based doses was a relative scaling, this renormalization was accomplished by multiplying the aperture dose by a simple scale factor. The same renormalization factor was used for all plans in each patient case, so that this did not confound any changes in dose resulting from the errors examined in the study. Throughout the study, the monitor units calculated by the optimization engine were used, without any application of a scale factor.

**Figure 3:**
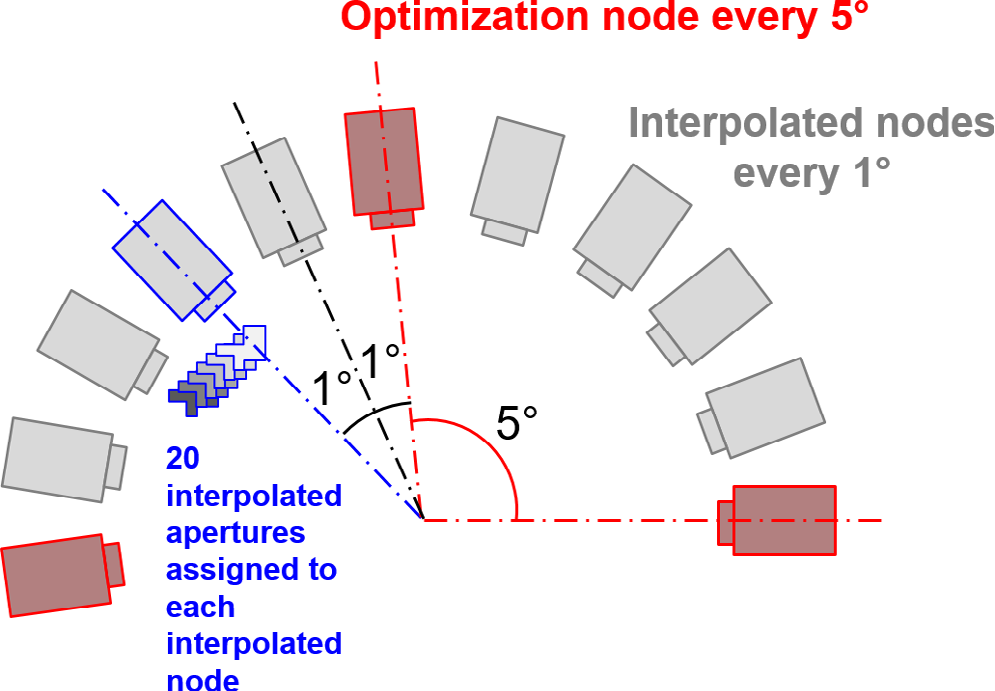
The model for control point interpolation. There are four interpolated nodes between each optimization node. The 20 interpolated apertures between each interpolated node allow for the modeling of multileaf collimator leaf motion and are all assigned to the following interpolated node. [Color figure can be viewed at http://wileyonlinelibrary.com]

In the final dose recalculation, additional nodes were used to model the robot orientation and MLC leaf position as accurately as possible. Four intermediate nodes were added between each pair of nodes, such that the additional nodes, together with the second of the original pair of nodes, formed a set of five interpolated nodes.^15^ A further 20 equally weighted interpolated apertures, allowing for MLC leaf motion, were calculated between each of the interpolated nodes, and dose was then calculated and summed for all of the interpolated apertures (Fig. 3). This detailed modeling of the MLC motion was found previously to be essential for accurate dose calculation with relatively large movements of small apertures.^26^

### 2.4. Uncertainty models

The uncertainty models are described below. The uncertainties were considered independently so as to determine their individual contributions to the delivery uncertainty.

#### 2.4.1. Dose rate uncertainty

**Figure 4:**
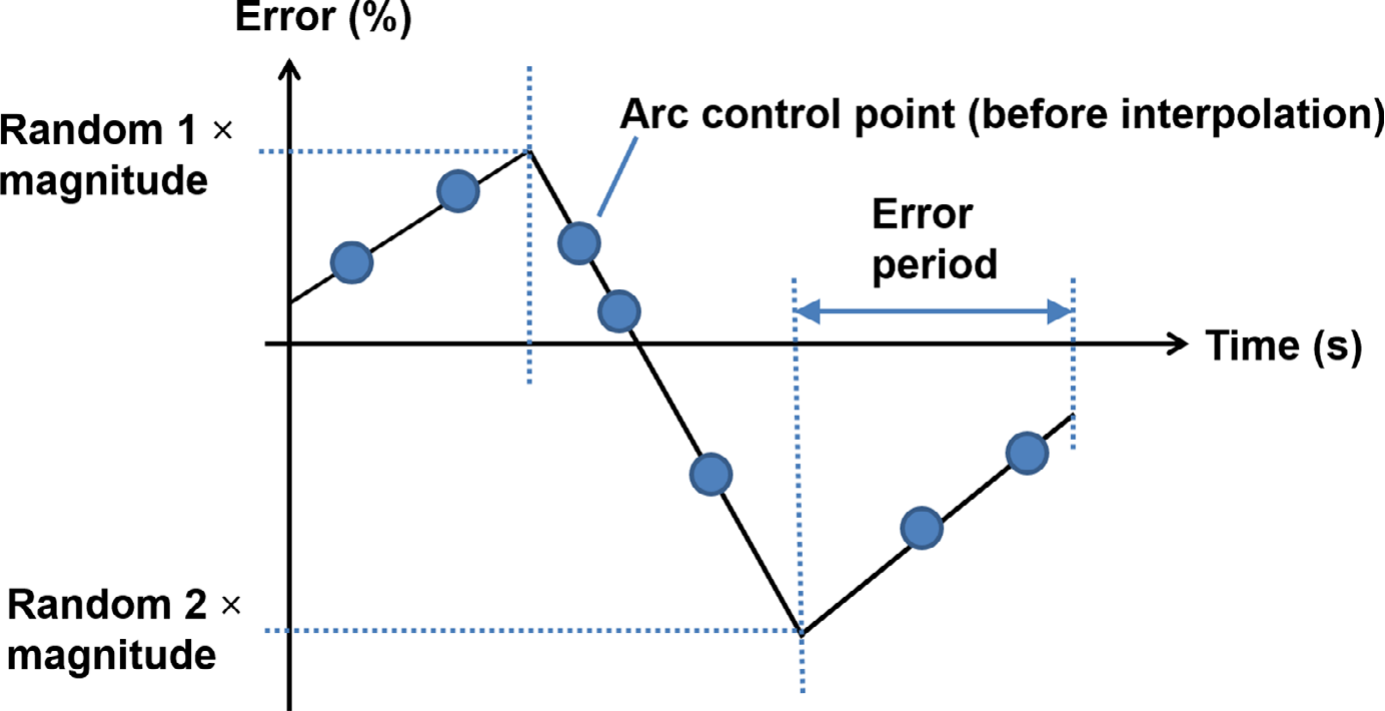
The dose rate uncertainty model. [Color figure can be viewed at http://wileyonlinelibrary.com]

Dose rate variations were modeled as a series of pseudorandom errors defined at integer numbers of the error period, which was set to 10, 30 or 60 s in turn (Fig. 4). The first of these values represented a rapidly drifting error and the last represented a slowly drifting error. The errors were sampled from a uniform distribution spanning from −*E* to +*E*, where *E* was the error magnitude, which was either 0.01 (i.e., 1%), 0.025 or 0.05. Between the error sampling points, the error was determined by linear interpolation. Large and abrupt changes (e.g. due to arcing) were ignored.

Addition of the random errors to the treatment arcs was carried out using the 104‐node optimized plans, before addition of interpolated nodes. The time, *t_n_*, at which segment *n* (*n* = 1…104) of the arc took place was calculated according to the formula:

(4) $$t_{n}=\sum_{i=1}^{n}\max\left(\frac{60}{D}M_{i},\;T_{R}\right),$$

where *M_i_* was the number of monitor units per fraction at node *i*, *D* = 1000 monitor units per minute was the maximum dose rate of the linear accelerator, and *T_R_* = 1.5 s was the robot traversal time. In other words, the time taken to deliver each segment of the arc was equal to the fixed traversal time *T_R_*, unless the monitor units, delivered at maximum dose rate, required additional time for delivery, in which case the time was equal to the time to deliver the monitor units. According to the time that a given control point was delivered, the appropriate value of error was determined from the error model. The monitor units for that control point were changed by the error:

(5) $$M_{E}=M_{i}+E\cdot M_{i},$$

where *M_i_* were the monitor units before addition of the error, *M_E_* were the monitor units after addition of the error, and *E* was the error. Strictly speaking, the delivery of *M_E_* monitor units took a different length of time to delivering *M_i_* monitor units, thereby influencing the time at which the error, *E*, should be sampled, but this secondary effect was neglected. Using *M_E_*, five interpolated nodes, each with 20 interpolated apertures, were calculated as described above. The three error periods and three error magnitudes gave nine combinations of error. For each combination of error period and magnitude, the calculation was repeated 10 times to give an indication of the spread of outcomes resulting from the selection of random errors.

#### 2.4.2. MLC position uncertainty

To model MLC leaf position uncertainty, at each control point of the arc (before interpolation), the position of each MLC leaf was moved 0.25 mm towards its position at the previous control point. The MLC leaf positions and the movement of 0.25 mm were defined at the nominal source‐axis distance of 800 mm. If the position of the MLC leaf at the previous control point was <0.25 mm away from its position at the current control point, the leaf was only moved as far as the position at the previous control point. This limitation occurred for between 4% and 13% of leaf movements, depending on the patient case, when considering all MLC leaves at all control points and neglecting closed leaf pairs. No change in position occurred for the first control point in the arc. The direction of MLC perturbation (i.e., use of a lag in position) and the representative magnitudes of uncertainty were based on worst‐case observations of the physical Cyberknife device.

After the change in position had been effected, five interpolated nodes, each with 20 intermediate apertures, were introduced and the dose was calculated, based on the interpolated plan. The experiment was repeated with a lag of 0.5 mm.

#### 2.4.3. Robot pointing uncertainty

**Figure 5:**
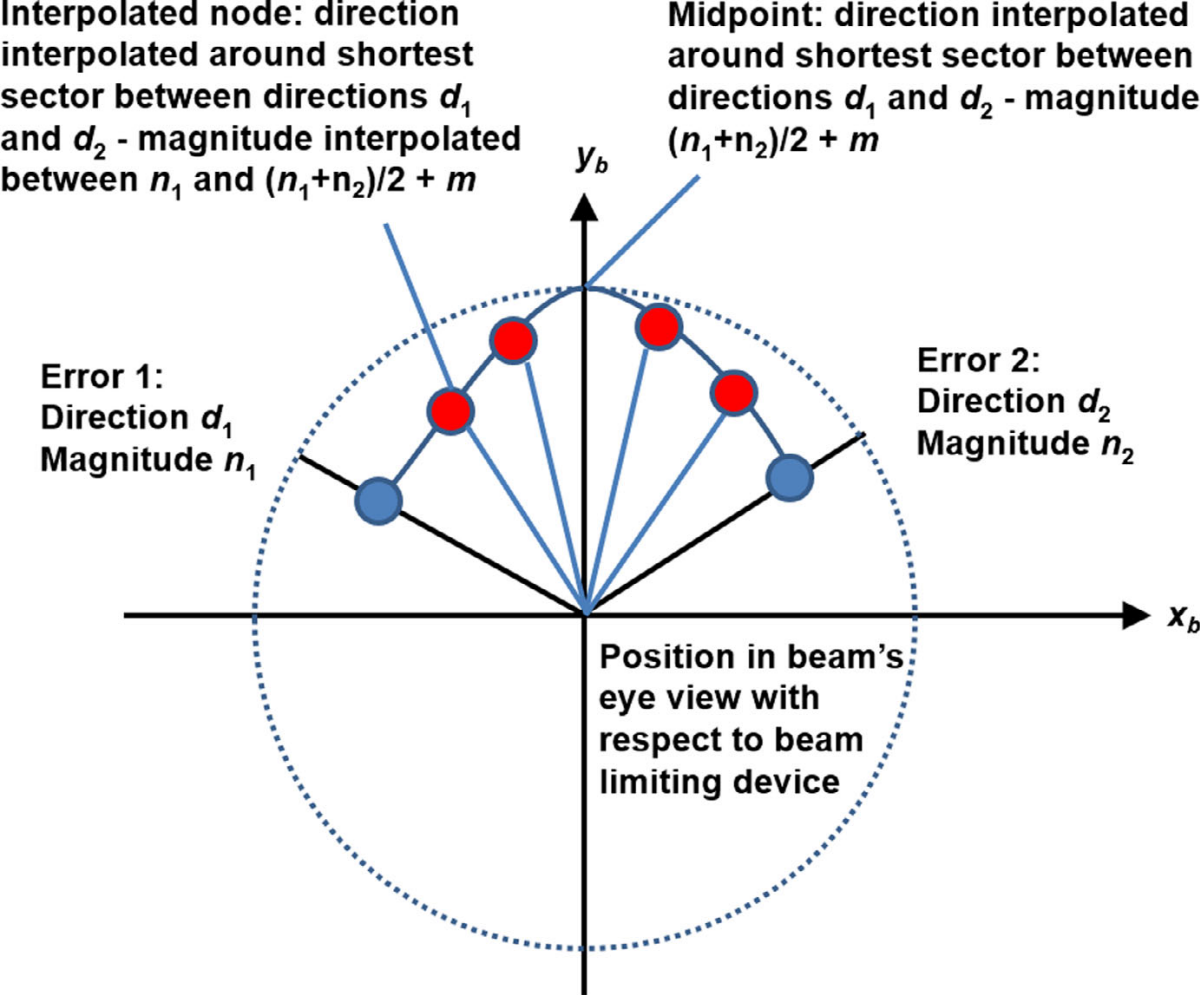
Model for robot pointing uncertainty. The axes are labeled according to IEC 61217 convention and the directions are defined relative to the −*y_b_* axis. [Color figure can be viewed at http://wileyonlinelibrary.com]

Each robot position was specified by a source point and target point, the target point corresponding to the isocenter on a conventional linear accelerator, but in this case varying in source‐target distance. To model the uncertainty in robot pointing, at each control point of the arc (before interpolation), the target point of the node was moved according to a uniformly sampled random direction with respect to the multileaf collimator. The error directions of the interpolated control points were then computed by linear interpolation between these directions. The interpolated directions were chosen to take the shortest route around the error circle (Fig. 5).

The magnitude of the error at the nodes before interpolation was uniformly sampled from 0…*N*, where *N* was either 0 or 1 mm. It was expected that the positioning of the robot would be less accurate between nodes than at the actual node positions, since between nodes, the control system would be carrying out some form of interpolation. An additional uniformly sampled error of 0…*M* was therefore added midway between the nodes, to reflect the additional uncertainty between nodes, where *M* was either 0.5 mm or 1.0 mm. The total error was thus (*n*_1_ + *n*_2_)/2 + *m* at the mid‐point between the nodes, where *n*_1_ and *n*_2_ were randomly sampled from a uniform distribution of width *N* and *m* was randomly sampled from a uniform distribution of width *M*. The magnitude of the error between the original nodes and the midpoint between them was calculated by linear interpolation, so that the total error changed smoothly from *n*_1_ to (*n*_1_ + *n*_2_)/2 + *m* and then smoothly to *n*_2_.

#### 2.4.4. Combination of uncertainties

The above uncertainties were applied individually to the treatment plans, but their impact if they were all present simultaneously was also investigated. A dose rate uncertainty of magnitude 0.025 with a time period of 30 s, an MLC lag of 0.25 mm and a robot pointing uncertainty of 1.0 mm at nodes with an additional uncertainty of 0.5 mm between nodes was used for this investigation. All of the uncertainties were applied to the same treatment plan and the effect on the dose distribution was observed. Three runs were carried out to assess the impact of the random variations.

A summary of the numerical values of uncertainty used in the model, together with their sources, is given in Table I.

**Table 1:** Values and sources of uncertainty used in this work.

Uncertainty	Numerical values	Sources
Dose rate uncertainty	1%, 2.5%, or 5% random variations over a time period of 10, 30 or 60 s	Accuray suggested
MLC lag uncertainty	0.25 or 0.5 mm lag in MLC position at nominal source‐axis distance of 800 mm	Accuray internal observations
Robot pointing uncertainty	‐ Direction with respect to MLC: random between 0° and 360° ‐ Magnitude at nodes: 0.0 mm or random between 0.0 and 1.0 mm ‐ Additional magnitude between nodes: random between 0.0 and 0.5 mm or between 0.0 and 1.0 mm	Yang et al.^11^, ^12^ Wang et al.^13^ Wang and Nelson^14^
Combination	‐ 2.5% random variation of dose rate over a time period of 30 s ‐ 0.25 mm lag in MLC position at nominal source‐axis distance of 800 mm ‐ Robot pointing uncertainty random between 0.0 and 1.0 mm at nodes and additionally random 0.0 to 0.5 mm between nodes	All of the above

MLC, multileaf collimator.

## 3. Results

### 3.1. Optimized treatment plans

The calculated monitor units and estimated delivery times for the five arcing treatment plans are given in Table II. The estimated delivery times are calculated using Eq. (4) for the final node, and the corresponding times for static delivery are also shown. Representative dose distributions for the treatment plans without delivery uncertainties are shown in a previous study, together with more details of comparison with static delivery.^15^

**Table 2:** Total monitor units per fraction and estimated delivery time per fraction for the five patient plans in the absence of uncertainty. For comparison purposes, the estimated static delivery time is also included.

	MU per fraction	Estimated delivery time (s)	Estimated static delivery time (s)
Prostate A	4978	355	675
Prostate B	10 579	672	1025
Lung	4312	290	672
Liver	9368	584	965
Partial breast	2833	235	554

### 3.2. Dose rate uncertainty

**Figure 6:**
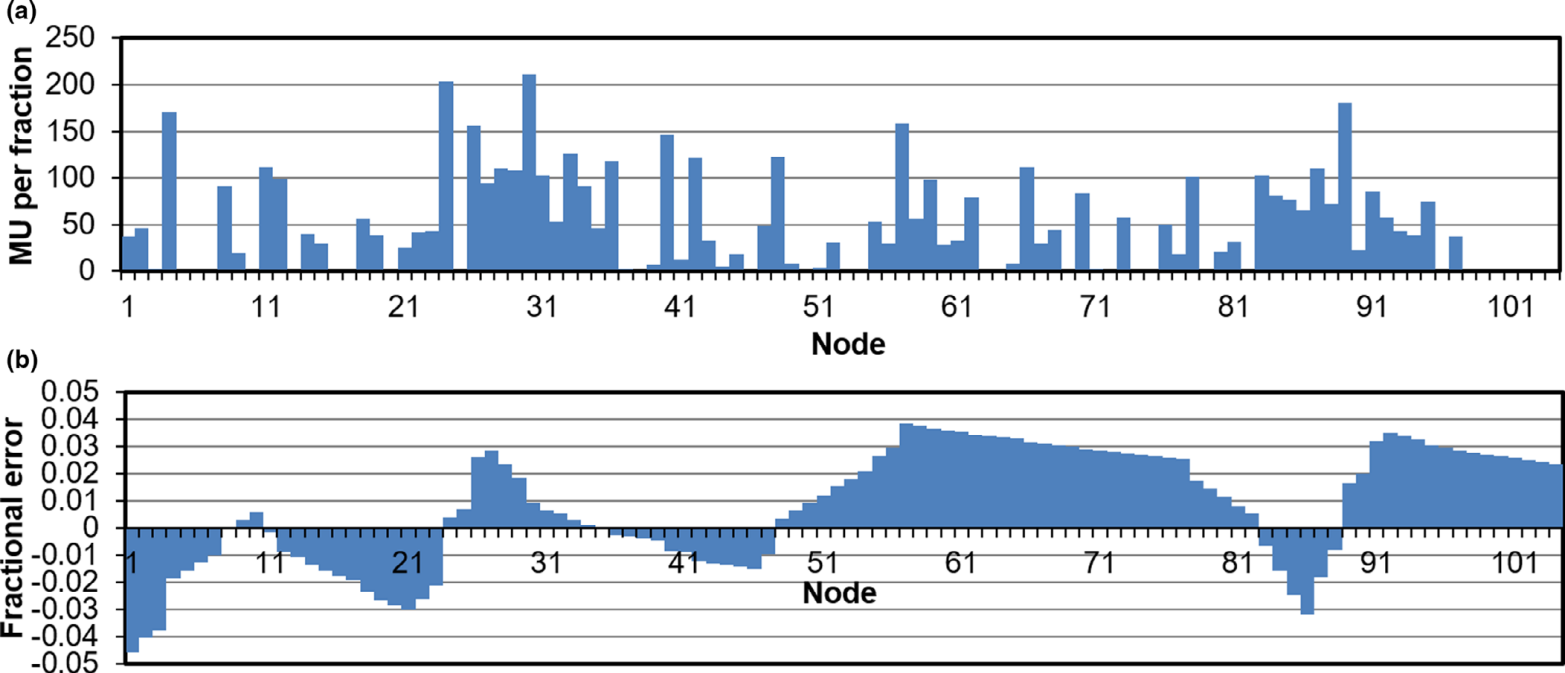
(a) Monitor units per fraction for prostate A as a function of delivery node. (b) The fractional error applied to the monitor units, using an error magnitude of 5% and a time period of 30 s. [Color figure can be viewed at http://wileyonlinelibrary.com]

**Figure 7:**
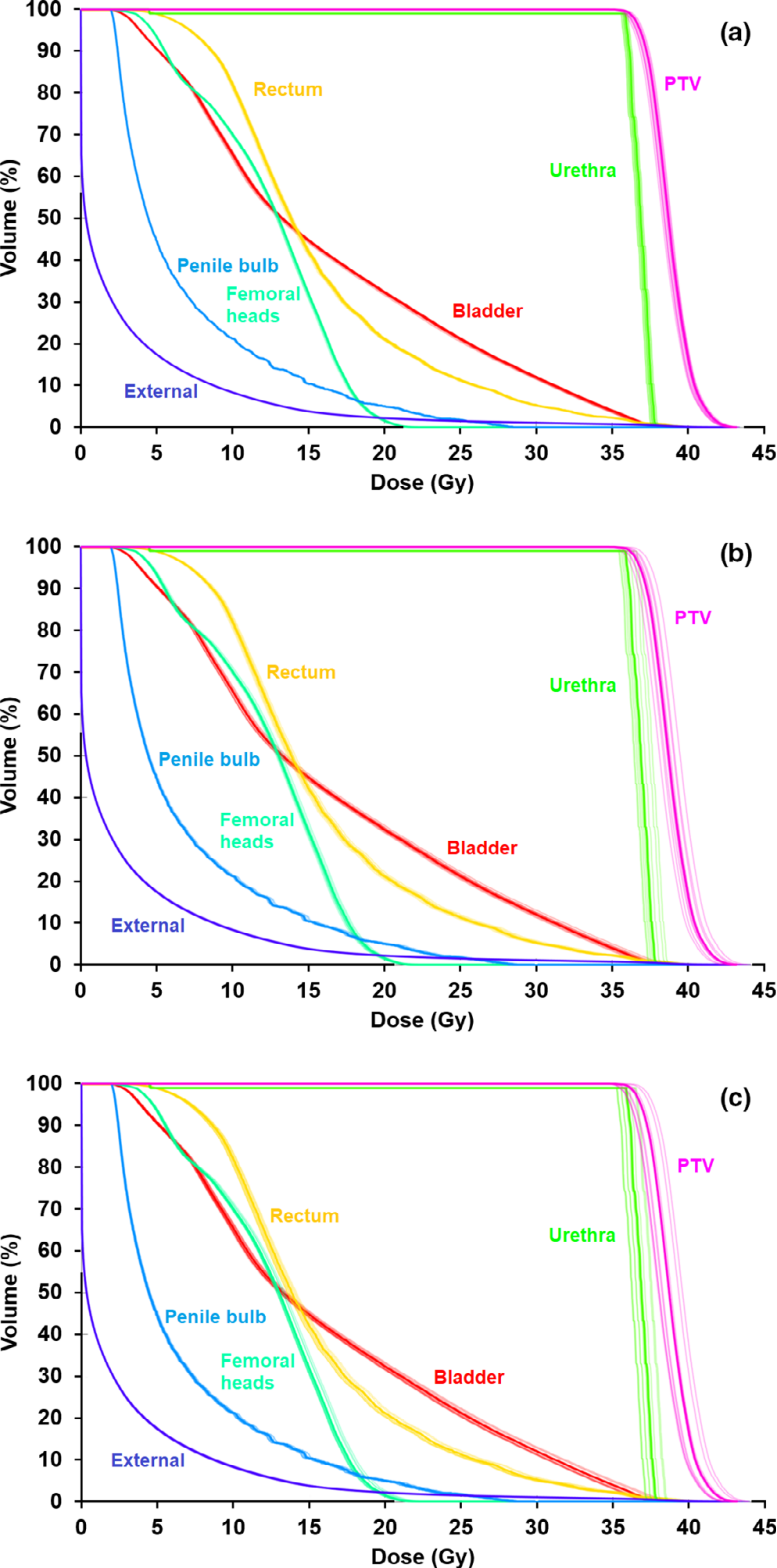
Dose‐volume histograms for prostate A for ten calculations, with error uncertainty of 5% using a time period of (a) 10 s, (b) 30 s and (c) 60 s. The bold lines represent the dose‐volume histograms without uncertainty. [Color figure can be viewed at http://wileyonlinelibrary.com]

The variation in monitor units per fraction with control point for the prostate A case, and the random uncertainty from the uncertainty model, are shown in Fig. 6. The error distribution [Fig. 6(b)] varies from run to run, due to the selection of a different set of random numbers each time. The results of 10 runs of the calculation are shown in Fig. 7 for 5% uncertainty at time periods of 10, 30, and 60 s. The graphs show a range of doses to the PTV, and this also occurs for the other patients. The statistics for the runs giving the lowest and highest doses are shown for all patients in Table III with 5% uncertainty and a time period of 60 s, that is, for the situation in Fig. 7(c).

**Table 3:** Planning target volume (PTV) statistics for the runs giving the lowest and highest PTV doses with 5% dose rate uncertainty and a time period of 60 s [see Fig. 7(c)].

Case	Run	PTV mean dose (Gy)	PTV min dose (Gy)	PTV max dose (Gy)
Prostate A	No error	38.81	35.35	43.26
	Minimum	38.11	34.70	42.53
	Maximum	39.69	36.19	44.08
Prostate B	No error	54.42	29.50	93.65
	Minimum	53.36	28.98	92.00
	Maximum	55.39	30.03	95.32
Lung	No error	55.83	45.46	62.42
	Minimum	54.52	44.36	60.99
	Maximum	56.72	46.16	63.43
Liver	No error	51.01	40.05	59.90
	Minimum	49.91	39.26	58.66
	Maximum	52.12	40.77	61.39
Partial breast	No error	39.01	32.48	45.23
	Minimum	37.93	31.66	43.91
	Maximum	39.55	33.04	45.82

**Figure 8:**
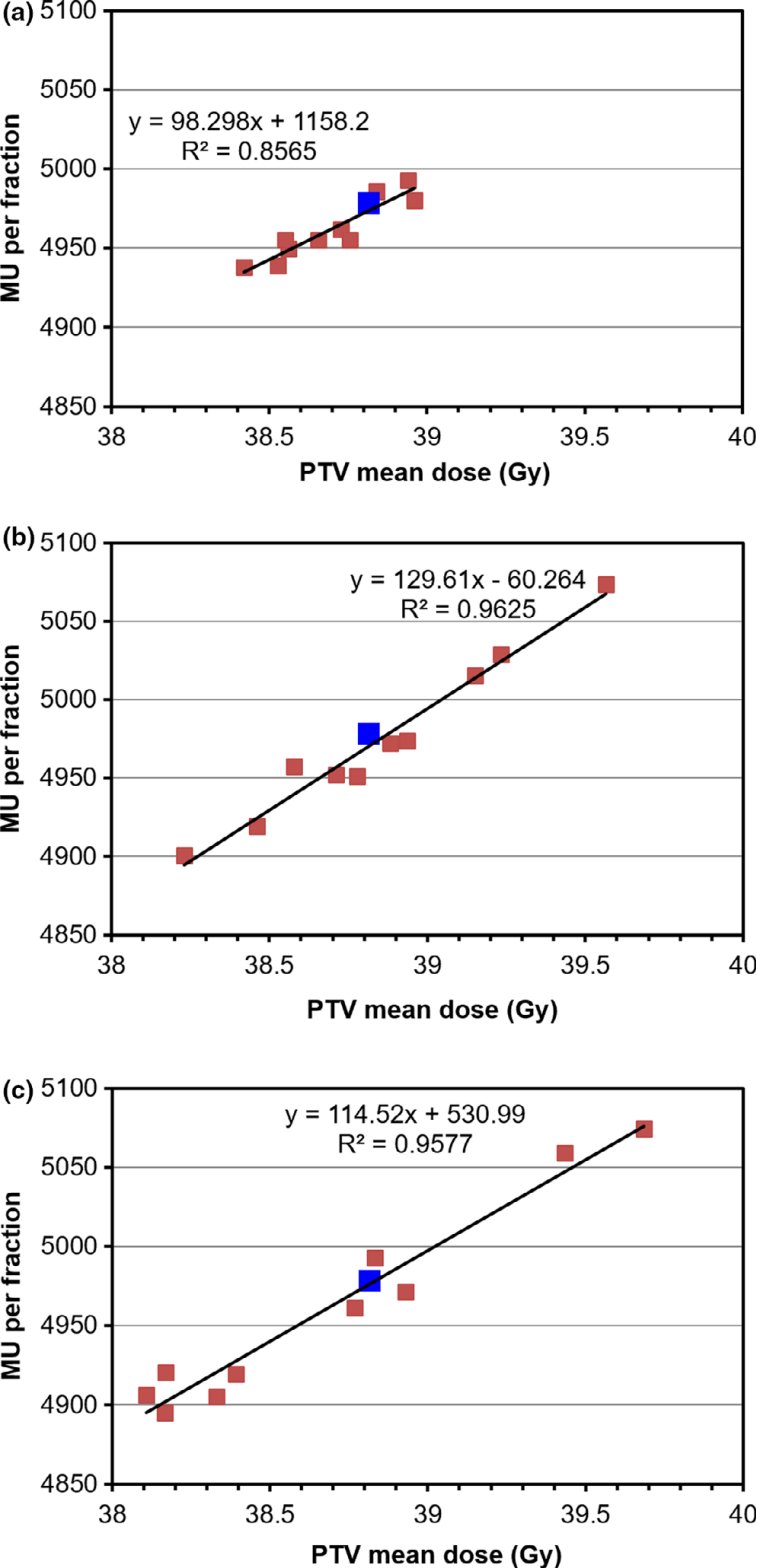
Correlation between planning target volume mean dose and total monitor units per fraction for prostate A for ten calculations, with error uncertainty of 5% using a time period of (a) 10 s, (b) 30 s, and (c) 60 s. The blue square represents the plan without uncertainty and the line shows a linear fit to the data. [Color figure can be viewed at http://wileyonlinelibrary.com]

The variation in PTV dose is accompanied by a variation in total monitor units per fraction. The predominant effect of the random variation of the monitor units is to change the total number of monitor units delivered, and this correlates closely with the variation in dose to the PTV (Fig. 8). The longer time period results in fewer random samples of error over the length of the delivery, so that the errors are less likely to cancel than with many samples. Consequently, the longer time period gives rise to a larger fluctuation in PTV dose than with the shorter time period.

The other error magnitudes of 0.01 and 0.025 give similar results to an error magnitude of 0.05, but with a correspondingly scaled down effect. The standard deviation of the error is expected to be proportional to the error magnitude, so a curve of the form $\sigma =M\sqrt{aT^{2}+bT}$ is fitted to the graphs of standard deviation vs error magnitude, where *σ* is the standard deviation, in Gy, of the PTV mean dose, *M* is the magnitude of the dose rate error, as a dimensionless proportion of the correct dose rate, and *T* is the time period, in seconds, of the dose rate variation. The values of *a* and *b* are constant and to be determined. By first plotting *σ* against *M* for several values of *T* and then fitting a polynomial of the form $aT^{2}+bT$ to a plot of the squared gradient of these curves against *T*, the behavior is found to be modeled by the equation:

(6) $$\sigma =M\sqrt{0.0018T^{2}+1.9084T}.$$

**Figure 9:**
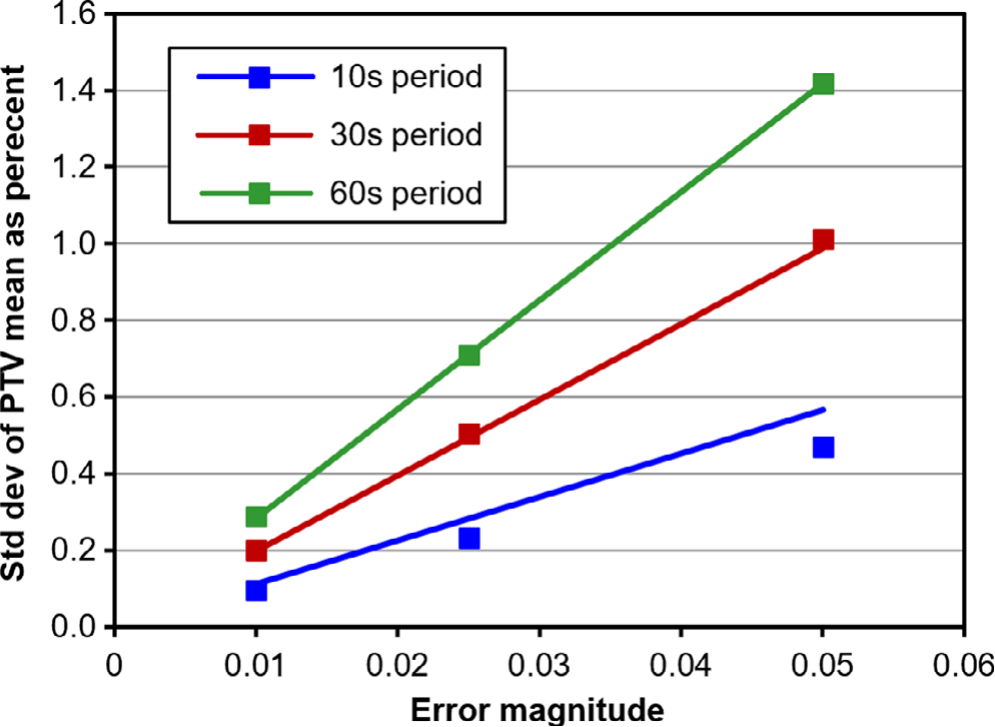
Standard deviation of planning target volume (PTV) mean dose as a function of the magnitude and period of dose rate error for the prostate A case. The standard deviation of PTV mean dose is expressed as a percentage of the PTV mean dose for the plan without errors. The squares show the observed standard deviation of PTV mean dose, and the lines show the result of using Eq. (7). [Color figure can be viewed at http://wileyonlinelibrary.com]

As a percentage of the PTV mean dose for the plan without errors, the relationship between standard deviation and error magnitude and period is:

(7) $$\sigma =M\sqrt{0.0121T^{2}+12.666T},$$

where *σ* is now the standard deviation of the PTV mean dose, as a percentage of the correct PTV mean dose. The overall variation in PTV mean dose is shown in Fig. 9 for the prostate A case, with the modeled results using this equation included. The results of applying Eq. (7) to all of the patient cases are shown in Table IV for the largest error magnitude.

**Table 4:** Standard deviation of planning target volume (PTV) mean dose as a function of dose rate error magnitude and error period. For all cases except the first, only the results for worst case error magnitudes and periods are shown. For each combination of magnitude and period the actual variation of PTV mean dose observed in 10 random plans is compared against the variation predicted by Eq. (7).

Case	Magnitude	Period (s)	Predicted % SD	Actual % SD
Prostate A	0.01	10	0.11	0.10
	0.01	30	0.20	0.20
	0.01	60	0.28	0.29
	0.025	10	0.28	0.23
	0.025	30	0.49	0.50
	0.025	60	0.71	0.71
	0.05	10	0.57	0.47
	0.05	30	0.99	1.01
	0.05	60	1.42	1.42
Prostate B	0.05	60	1.42	1.10
Lung	0.05	60	1.42	1.37
Liver	0.05	60	1.42	1.29
Partial breast	0.05	60	1.42	1.26

For context, the observed data are based on a sample size of 10, so the standard error of the actual standard deviation, *σ*, shown in Fig. 9 and Table IV is $\sigma /\sqrt{2\left(n-1\right)}\approx \sigma /4$. In other words, the inherent variability of the data is approximately one quarter of the standard deviations given.

### 3.3. MLC position uncertainty

**Figure 10:**
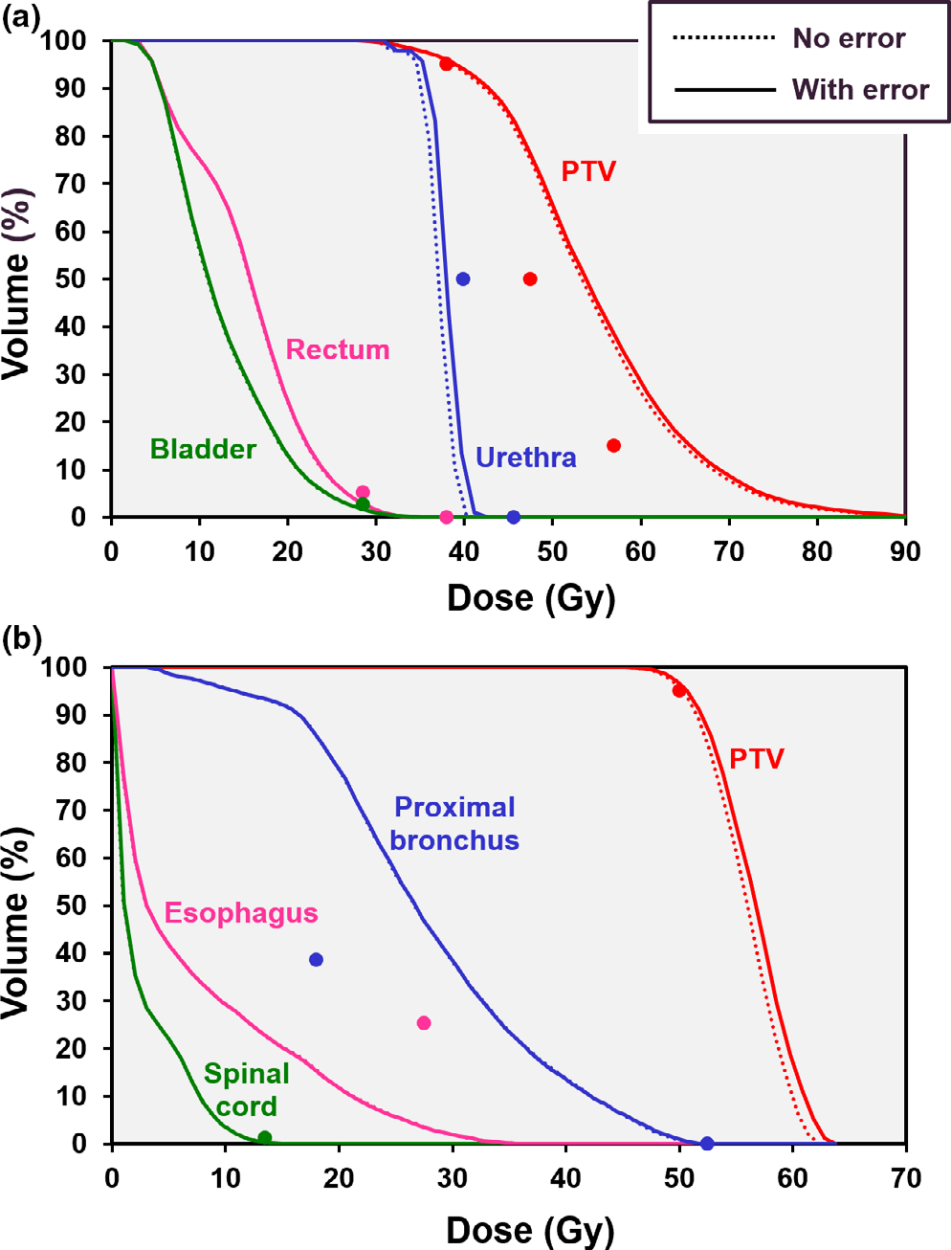
Dose‐volume histograms showing the impact of a 0.5 mm multileaf collimator (MLC) lag on the treatment plans for (a) prostate B case and (b) central lung case. Solid lines: with MLC lag, dotted lines: without MLC lag. The points represent the principal clinical constraints. [Color figure can be viewed at http://wileyonlinelibrary.com]

Dose‐volume histograms showing the impact of a 0.5 mm MLC lag are shown in Fig. 10 for the prostate B and lung cases. (Due to the large overlap of the PTV with the proximal bronchus in the lung case, the plan is unable to meet the constraint that the dose to 4 cm^3^ of proximal bronchus should not exceed 18 Gy.) The MLC lag has the effect of modifying the dose to the PTV by around 1%, with a slightly larger effect on the urethra in the prostate B case. Table V shows the statistics for all of the cases, for both 0.25 and 0.5 mm lag.

**Table 5:** Planning target volume (PTV) statistics as a function of multileaf collimator lag.

Case	Run	PTV mean dose (Gy)	PTV min dose (Gy)	PTV max dose (Gy)
Prostate A	No error	38.81	35.35	43.26
	0.25 mm lag	38.97	35.37	43.55
	0.5 mm lag	39.12	35.35	43.91
Prostate B	No error	54.42	29.50	93.65
	0.25 mm lag	54.73	29.51	94.88
	0.5 mm lag	54.97	29.44	95.59
Lung	No error	55.83	45.46	62.42
	0.25 mm lag	56.22	45.45	63.15
	0.5 mm lag	56.58	45.38	63.79
Liver	No error	51.01	40.05	59.90
	0.25 mm lag	51.18	39.92	60.36
	0.5 mm lag	51.35	39.83	60.82
Partial breast	No error	39.01	32.48	45.23
	0.25 mm lag	39.20	32.47	45.67
	0.5 mm lag	39.39	32.43	46.07

### 3.4. Robot pointing uncertainty

**Figure 11:**
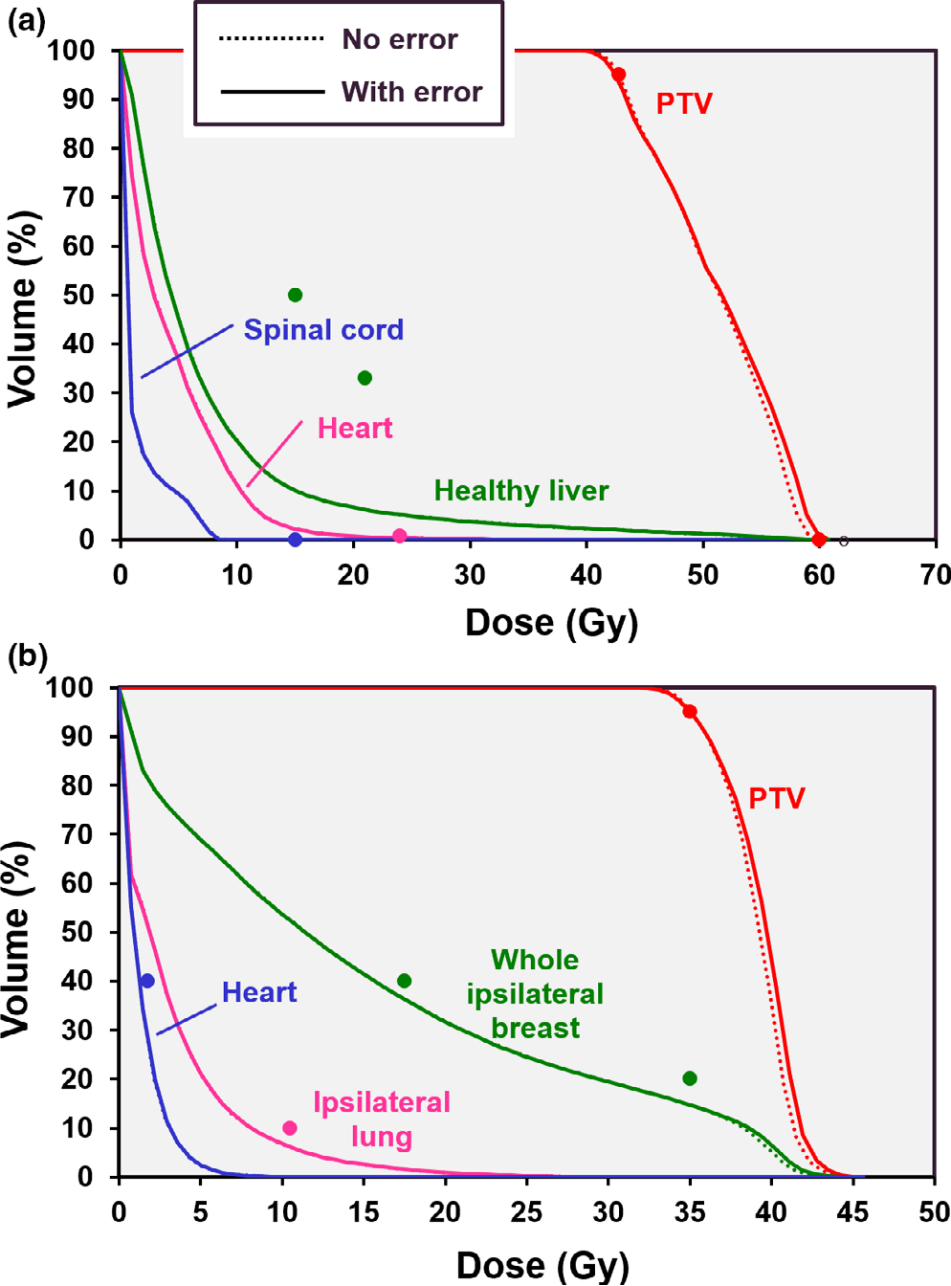
Dose‐volume histograms showing the impact of a robot pointing error of 1.0 mm at nodes and an additional error of 1.0 mm between nodes, for (a) liver case, and (b) partial breast case. Solid lines: with pointing error, dotted lines: without pointing error. The points represent the principal clinical constraints. [Color figure can be viewed at http://wileyonlinelibrary.com]

The effect of a randomly sampled robot pointing error of 1.0 mm at the delivery nodes, with an additional randomly sampled error 1.0 mm between the nodes is shown in Fig. 11 for the liver and partial breast cases. The effect of the error is to modify the PTV dose slightly, with an increase in dose usually being observed. Table VI shows the statistics for all cases and robot pointing errors.

**Table 6:** Planning target volume (PTV) statistics as a function of robot pointing error.

Case	Node error (mm)	Additional inter‐node error (mm)	PTV mean dose (Gy)	PTV min dose (Gy)	PTV max dose (Gy)
Prostate A	0.0	0.0	38.81	35.35	43.26
	0.0	0.5	39.14	35.35	44.02
	0.0	1.0	39.13	35.37	43.94
	1.0	0.5	39.12	35.35	43.97
	1.0	1.0	38.94	35.19	43.88
Prostate B	0.0	0.0	54.42	29.50	93.65
	0.0	0.5	54.97	29.53	95.58
	0.0	1.0	54.94	29.58	95.74
	1.0	0.5	54.95	29.21	95.45
	1.0	1.0	54.89	29.35	96.22
Lung	0.0	0.0	55.83	45.46	62.42
	0.0	0.5	56.61	45.44	63.77
	0.0	1.0	56.62	45.35	63.85
	1.0	0.5	56.54	45.54	63.51
	1.0	1.0	56.64	45.35	64.08
Liver	0.0	0.0	51.01	40.05	59.90
	0.0	0.5	51.35	39.86	60.80
	0.0	1.0	51.32	39.67	60.80
	1.0	0.5	51.30	39.82	61.02
	1.0	1.0	51.27	39.30	60.78
Partial breast	0.0	0.0	39.01	32.48	45.23
	0.0	0.5	39.37	32.50	46.04
	0.0	1.0	39.38	32.48	46.06
	1.0	0.5	39.34	32.22	46.23
	1.0	1.0	39.36	32.34	45.67

### 3.5. Combination of uncertainties

The effect of a dose rate uncertainty of magnitude 0.025, an MLC lag of 0.25 mm and a robot pointing uncertainty of 1.0 mm with an additional 0.5 mm between nodes, is to adjust the PTV mean dose by around 1%, mostly with the minimum and maximum doses following the trend of the mean dose. The results of three runs for each patient are shown in Table VII.

**Table 7:** Planning target volume (PTV) statistics for three runs with a combination of dose rate, multileaf collimator lag and robot pointing errors.

Case	Run	PTV mean dose (Gy)	PTV min dose (Gy)	PTV max dose (Gy)
Prostate A	No error	38.81	35.35	43.26
	Run 1	38.71	35.15	43.23
	Run 2	39.08	35.47	43.80
	Run 3	38.89	35.41	43.44
Prostate B	No error	54.42	29.50	93.65
	Run 1	54.48	29.68	94.23
	Run 2	54.76	29.43	94.62
	Run 3	54.51	29.66	94.45
Lung	No error	55.83	45.46	62.42
	Run 1	55.49	45.13	62.31
	Run 2	56.21	45.52	62.99
	Run 3	56.15	45.58	63.30
Liver	No error	51.01	40.05	59.90
	Run 1	50.84	39.83	60.02
	Run 2	51.33	39.90	60.92
	Run 3	51.05	39.73	60.03
Partial breast	No error	39.01	32.48	45.23
	Run 1	38.89	32.30	44.98
	Run 2	39.21	32.19	45.86
	Run 3	39.08	32.30	45.62

## 4. Discussion

The ability of the Cyberknife system to treat from a variety of noncoplanar beam directions^27^ is useful for high‐quality treatment of SBRT.^1^, ^2^, ^3^, ^4^ Treating from such a range of orientations is time‐consuming, so that the total treatment time is long. However, several recent studies have shown that use of a dynamic arc delivery can reduce the treatment time by a factor of approximately two. For example, Kearney et al.^10^ report for prostate and brain patients a speedup of 1.5 ± 0.3, depending on the parameters used by the optimizer, and Bedford et al.^15^ report a median speedup of 1.90 (range 1.53 to 2.36). Similarly, using a C‐arm linear accelerator, Wild et al.^24^ predict a delivery time of 6.5 min on average for noncoplanar VMAT. The choice of arc trajectory is likely to have an impact on the delivery time.^28^

Since the Cyberknife does not currently deliver radiation in dynamic mode, it is not possible to measure the dosimetric accuracy of dynamic delivery in the same way that is possible for a C‐arm linear accelerator.^28^ However, several recent studies have reported on the geometric accuracy of the device, which have been used in the present study to give estimates of the expected dosimetric performance.^11^, ^12^, ^13^, ^14^ Variations in dose rate are shown to have negligible effect on the shape of the dose distribution, but rather to impact on the total monitor units delivered, which in turn affect the scaling of the plan, most noticeably in the mean dose to the PTV. There is a corresponding effect on the organs at risk in proportion to the dose received by these organs at risk. For example, proximal structures such as the urethra and the proximal bronchus are impacted similarly to the PTV, whereas distal structures are hardly affected at all. The magnitude of the dosimetric error determined in this study is likely to be a worst‐case estimate, as the variation in the monitor units produced by the optimizer is quite high (Fig. 6), with some nodes not delivering any dose at all.

The impact of an MLC lag is shown to cause a small change in PTV dose. This is almost always in the form of an increase in PTV dose of around 1% for a 0.5 mm lag in MLC leaf position. This is in accord with the work of Christiansen et al.,^25^ who demonstrate large changes in PTV dose depending upon whether MLC leaves are considered static at their positions while passing the delivery control points, or dynamic with motion between control points. The present work uses a dynamic beam model throughout, in order to model the dose delivery as accurately as possible, but the comparison of Christiansen et al.^25^ with a static situation is very similar to a situation with a severe MLC lag.

In terms of practical measurements on the current generation of Cyberknife, perhaps the most that is known is for the accuracy of robot pointing at static nodes.^11^, ^12^ The effect of a random error in the order of 1.0 mm at control points, with an additional error between control points, is to modify the PTV dose by approximately 1%. An uncertainty in robot position is similar in nature to an uncertainty in MLC leaf position, so the similarities between the results for MLC lag and robot position are rational. The uncertainty in robot pointing is modeled in this study as a shift in the target point, with the robot itself positioned correctly (but not orientated correctly). The results might be affected by using a model in which the robot itself is positioned incorrectly. However, Wang and Nelson^14^ show that the overall result of translation and rotation errors is a shift in the target point of less than 1 mm, so the model used in the present study is representative.

Taking these results together, the study shows that the Cyberknife is expected to be able to deliver dynamic arc beams with a dosimetric accuracy of the order of 1‐2%. This is comparable to accuracy studies for Cyberknife with static beam delivery, on the one hand, and conventional linear accelerators delivering VMAT, on the other hand. For Cyberknife with static beam delivery, Dieterich et al.^29^ and Moore et al.^30^ recommend a dosimetric accuracy of 90% within 2% and 2 mm in comparison with the treatment planning system. Some of this tolerance is used by the treatment planning system, but the accuracy predicted by the present study is within this order of magnitude. Similarly, for coplanar VMAT delivery,^31^, ^32^, ^33^ Ling et al.^34^ report MLC leaf positions generally within 0.5 mm of their expected position, and relative dose accuracy of 0.7%. Bedford and Warrington^35^ report a dynamically delivered dose generally within 2% of the same plan delivered statically, and a gamma agreement of 3% and 3 mm for delivery of a complete treatment plan. Mans et al.^36^ recommend a dose rate dependence with gantry angle of better than 0.5% and MLC leaf positioning of better than 1.0 mm and preferably better than 0.5 mm. For comparison with a treatment planning system, 90% of the delivered dose should be within 3% and 3 mm.

The results have been obtained for PTV volumes between 14 cm^3^ (lung case) and 113 cm^3^ (prostate A case). A larger relative impact is observed with MLC position uncertainty for the lung case than the prostate A case, which may be partly due to the difference in volume. It is very likely that different results may occur for very small stereotactic volumes below 10 cm^3^, in which case the present results should be treated with caution.

The choice of arc trajectory may have some impact on the dosimetric accuracy. The trajectory used in this study is the result of a heuristic which aims to provide a uniform coverage of the beam orientation space, while taking into account cabling requirements and collision avoidance. This trajectory is asymmetric, and has been shown in a previous study^15^ to compare well with the standard (symmetric) body path for the Cyberknife in terms of dose distribution and conformity. The trajectory affects the number of monitor units, the MLC shapes and the robot positions, and is therefore likely to affect the delivery uncertainties and hence the dosimetric results of the study to some degree. However, it is thought that the principal conclusions of this study are likely to be similar to whichever trajectory is chosen.

Further improvement in the delivery accuracy could be obtained by the use of robust treatment planning.^37^ Although this is usually applied to overcome uncertainties in target position, setup uncertainties, or range uncertainties, it could be used to overcome the limitations of linear accelerator performance. By informing the optimization engine of the possible scenarios that can occur during treatment delivery, the treatment plan can be designed to give an acceptable dose distribution in most or all situations. For this reason, models of treatment delivery accuracy are important.

## 5. Conclusions

Estimated uncertainties in dose rate, MLC leaf position, and robot target position are shown to have a dosimetric impact of around 1–2% during the arc delivery of SBRT using Cyberknife. The dose rate uncertainty affects the total number of monitor units delivered, which in turn affects the delivered dose. A lag of around 0.5 mm in MLC leaf position affects the delivered dose by approximately 1%, usually with an increase in dose, and an uncertainty in robot pointing has a similar effect. The dose to the PTV is affected most, with a lesser impact on organs at risk that receive less dose in the treatment plan.

## Conflicts Of Interest

This work has been funded by Accuray Inc.
